# Association Between Interpregnancy Interval and Risk of Preterm Birth and Its Modification by Folate Intake: The Japan Environment and Children’s Study

**DOI:** 10.2188/jea.JE20210031

**Published:** 2023-03-05

**Authors:** Kanami Tanigawa, Satoyo Ikehara, Meishan Cui, Yoko Kawanishi, Tadashi Kimura, Kimiko Ueda, Kazumasa Yamagishi, Hiroyasu Iso

**Affiliations:** 1 Public Health, Department of Social Medicine, Osaka University Graduate School of Medicine, Osaka, Japan; 2 Department of Obstetrics and Gynecology, Osaka University Graduate School of Medicine, Osaka, Japan; 3 Osaka Maternal and Child Health Information Center, Osaka Women’s and Children’s Hospital, Osaka, Japan; 4 Department of Public Health Medicine, Faculty of Medicine, and Health Services Research and Development Center, University of Tsukuba, Ibaraki, Japan

**Keywords:** interpregnancy interval, preterm birth, folate, folic acid, prospective studies

## Abstract

**Background:**

Both short and long interpregnancy intervals (IPIs) have been associated with risk of preterm birth, but the evidence is limited in Asians. It is also uncertain whether the association is modified by dietary folate intake or folic acid supplementation during pregnancy. Thus, we examined associations between IPI and risk of preterm birth and effect modification of those associations by dietary intake of folate and supplementation with folic acid on the basis of a nationwide birth cohort study.

**Methods:**

Among 103,062 pregnancies registered in the Japan Environment and Children’s Study, 55,203 singleton live-birth pregnancies were included in the analysis. We calculated IPI using birth date, gestational age at birth of offspring, and birth data of the latest offspring. Odds ratios (ORs) and 95% confidence intervals (CIs) of the risk of preterm birth were estimated according to IPI categories.

**Results:**

Both <6-month and ≥120-month IPIs were associated with an increased risk of preterm birth, compared with an 18–23-month IPI. The multivariable ORs were 1.63 (95% CI, 1.30–2.04) for <6-month and 1.41 (95% CI, 1.11–1.79) for ≥120-month IPIs. These associations were confined to women with inadequate intake of dietary folate and folic acid supplementation during pregnancy. Multivariable ORs were 1.76 (95% CI, 1.35–2.29) for <6-month IPI and 1.65 (95% CI, 1.24–2.19) for ≥120-month IPI.

**Conclusion:**

Both <6-month and ≥120-month IPIs were associated with an increased risk of preterm birth. These higher risks were confined to women with inadequate intake of dietary folate and folic acid supplementation during pregnancy.

## INTRODUCTION

Preterm birth is recognized as a worldwide public health problem in terms of survival and quality of life. The World Health Organization (WHO) reported that approximately 15 million infants were born preterm in 2014.^1^ In addition, 1 million children under 5 years old died related to preterm birth complications in 2015.^2^

Interpregnancy interval (IPI) is defined as the interval between the birth date of the latest delivery and conception of the next pregnancy.^3^ Both short and long IPIs have been associated with increased risks for preterm birth in the previous studies reported from the United States, Latin America, and Europe,^4^^–^^8^ but the evidence is limited in Asians. Because IPI has become shorter rapidly in Japan since the early 2000s,^9^ there is a need to consider this association. Nevertheless, there is only one report of the association between IPI and preterm birth in Japanese women.^10^ In that retrospective cohort study of 547 women with a previous history of preterm birth, an IPI of <12 months was associated with 2.1-fold higher risk of recurrent preterm birth than that with an IPI ≥12 months.^10^

There is limited evidence regarding modification of risk of preterm birth related to IPI; however, dietary intake of folate and supplementation with folic acid during pregnancy may be potential protective factors for perinatal outcomes related to IPI.^11^ A short IPI without folic acid supplementation was associated with a lower birthweight and higher risk of small size for gestational age^12^; however, it remains uncertain as to whether the association between IPI and risk of preterm birth is modified by dietary intake of folate or supplementation with folic acid during pregnancy. Therefore, we evaluated the associations of IPI with risk of preterm birth and the modification of this association by dietary intake of folate and supplementation with folic acid in a large birth cohort study from Japan.

## METHODS

### Study population

The Japan Environment and Children’s Study (JECS) is a nationwide birth cohort study funded by the Ministry of the Environment, Japan. Pregnant women were recruited between January 2011 and March 2014 from medical facilities or local municipal offices. A total of 103,062 pregnancies were confirmed. The details of the study are described elsewhere.^13^^,^^14^ The protocol of this study was approved by the Ministry of the Environment’s Institutional Review Board on Epidemiological Studies (date of approval: August 9 2010 and approval number: 2010-2R) and by the Ethics Committees of all participating institutions. The JECS obtained written informed consent from all participants.

**Figure 1.**
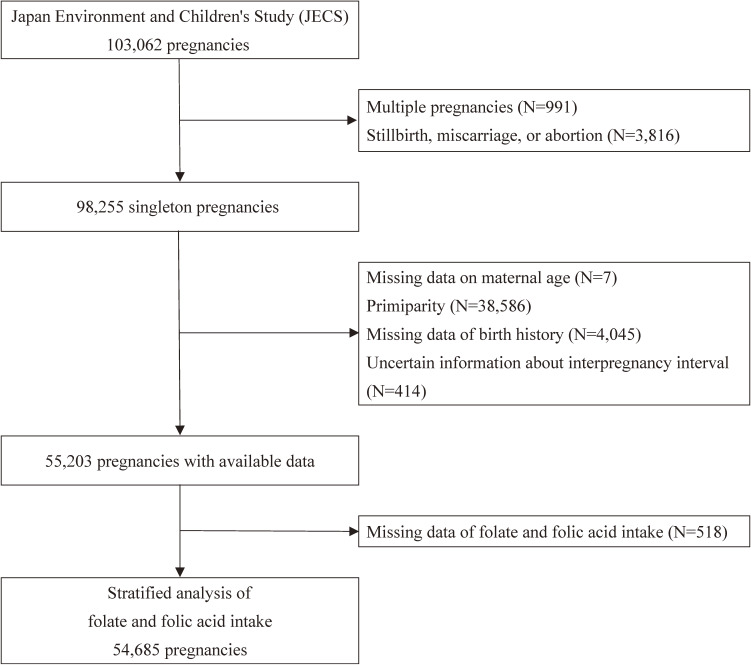
Flow chart of participant selection.

Among 98,255 singleton livebirth pregnancies, we excluded 7 individuals with missing maternal age, 38,586 individuals because of primiparity, 4,045 individuals with missing birth history, and 414 individuals who had uncertain information regarding IPI. Finally, 55,203 singleton livebirth pregnancies were included in the present analysis (Figure 1).

### Data collection

We distributed self-administered questionnaires at registration and during the second/third trimester. Questions included maternal demographic characteristics, socioeconomic status, lifestyle, mental health, and birth date of siblings. Data on the history of use of anemia medication before and during pregnancy were obtained during interviews at registration and during the second/third trimester. Maternal anthropometric data before and during pregnancy and data on complications before and during pregnancy, maternal age at delivery, number of births, birth dates of offspring, gestational age, and perinatal outcomes were obtained from medical records provided by the participants’ obstetricians. Body mass index (BMI) before pregnancy was calculated as weight before pregnancy (kg)/height^2^ (m^2^).

### Exposure and outcome

IPI is the interval between the birth date of the latest delivery and conception of the next pregnancy.^3^ We defined IPI as the interval between birth date of the latest sibling and the conception of pregnancy registered in the JECS. We converted gestational age from weeks into months and calculated IPI by subtracting the gestational age (in months) at birth of the offspring registered in the JECS from the interval between the date of the two deliveries (in months).^3^ IPI was categorized as <6, 6–11, 12–17, 18–23, 24–29, 30–35, 36–41, 42–47, 48–53, 54–59, 60–89, 90–119, or ≥120 months.

Preterm birth was defined as delivery at <37 weeks of gestation, based on medical record transcripts. Gestational age was estimated clinically from the date of last menstrual period, by measuring the crown–rump length, or from the date of in vitro fertilization.

### Intake of dietary folate and folic acid supplementation

Dietary intake of folate during pregnancy was calculated from the food frequency questionnaire (FFQ)^15^ administered during the second/third trimester. In the FFQ, we estimated daily dietary intake by multiplying frequency by the standard portion size of food items, on the basis of the Japanese Food Composition Tables, 5^th^ Revision.^16^ Spearman’s correlation coefficient comparing FFQ and 12-day food records over four seasons at intervals of each 3 months was 0.62 for dietary intake of folate.^15^

Information about supplementation with folic acid during pregnancy was included in the questionnaire and in FFQ administered during the second/third trimester. In that questionnaire, we asked about the frequency of supplementation with folic acid: ≥2 times/day, once/day, 4–6 times/week, 1–3 times/week, 2–3 times/month, once/month, or never. In the FFQ administered during the second/third trimester, if participants used supplements during pregnancy, they wrote down the trade names or types of supplements used and selected the category for the frequency of use of each supplement: ≥4 times/day, 2–3 times/day, once/day, 5–6 times/week, 3–4 times/week, or 1–2 times/week. When participants answered the frequency of folic acid supplementation as once or more times/day in the questionnaire or FFQ during the second/third trimester, we defined these cases as daily supplementation of folic acid.

Furthermore, we defined dietary intake of ≥400 µg/day of folate and/or daily supplementation with folic acid as adequate intake during pregnancy. We defined folate dietary intake <400 µg/day and no daily supplementation of folic acid as inadequate intake during pregnancy. This is because Japanese pregnant women are recommended to take at least 400 µg/day folate as part of their diet during the second/third trimester,^17^ and most folic acid supplements in Japan contain ≥400 µg/day folic acid.^18^ Bioavailability of folate is 50 percent lower than that of folic acid.^19^

### Statistical analyses

The present analysis is based on the dataset jecs-an-20180131, which was released in March 2018. We calculated means and prevalence of characteristics according to IPI. Test for trends were performed modeling with the median value in each category of IPI. Multivariable logistic regression models were used to calculate the maternal age-adjusted and multivariable odds ratios (ORs) and their corresponding 95% confidence intervals (CIs) for the association between IPI and the risk of preterm birth. The reference category of IPI was defined as 18–23 months, because WHO recommends that the next pregnancy be avoided for at least 18 months from birth.^20^ In the multivariable model, we adjusted for maternal age at delivery (continuous) and potential confounders including residential area (15 regional centers), maternal BMI before pregnancy (quintile), maternal education levels (junior high school, high school, technical college/vocational school/junior college, or university/graduate school), maternal smoking during pregnancy (non-smoker, ex-smoker, or current smoker), frequency of maternal passive smoking during pregnancy (almost never, once, 2–6 days, or every day per week), maternal drinking during pregnancy (non-drinker, ex-drinker, or current drinker), maternal physical activity during pregnancy (The short version of the International Physical Activity Questionnaire [IPAQ]^21^^,^^22^; quintiles), household income during pregnancy (<2, 2–3.9, 4–5.9, 6–7.9, or ≥8 million yen/year), maternal occupation during pregnancy (full-time worker, self-employed worker, dispatched worker, part-time worker, housewife, or other), marital status during pregnancy (married, unmarried, or divorced/widowed), number of children (1, 2, or ≥3 children), maternal stress during pregnancy (Kessler Psychological Distress Scale [K6]^23^: <5, 5–12, or ≥13 points), maternal dietary intake of iron during pregnancy (quintiles), maternal use of anemia medication during pregnancy (yes or no), maternal dietary intake of folate and supplementation with folic acid during pregnancy (inadequate intake of dietary folate and folic acid supplementation during pregnancy or adequate intake of dietary folate and/or folic acid supplementation during pregnancy), the latest pregnancy outcome (livebirth or stillbirth/miscarriage/abortion), spontaneous pregnancy (yes or no), previous history of preterm birth (yes or no), maternal medical history of hypertensive disorder of pregnancy (yes or no), and gestational diabetes (yes or no). Missing data for confounding factors were included as categorical variables in the model. The proportions of missing values were 7.4% for household income during pregnancy, 6.4% for previous history of preterm birth, 5.8% for maternal physical activity during pregnancy, and 0 to 1.8% for other confounding factors.

We measured the interaction between IPI and folate/folic acid intake on the risk of preterm birth, and a stratified analysis was conducted according to folate and/or folic acid intake. We divided dietary intake of folate and supplementation with folic acid during pregnancy into two categories (adequate or inadequate) (*N* = 54,685).

All statistical tests were two-tailed, and *P* < 0.05 was considered statistically significant. All analyses were conducted using SAS software version 9.4 (SAS Institute, Inc., Cary, NC, USA).

## RESULTS

Table 1 shows the baseline characteristics of the participants in terms of IPI; 2.9% of women had <6-month IPIs and 2.4% had ≥120-month IPIs. Compared to those women with <6-month IPI, those with longer IPIs were older, were younger at first birth, reported less physical activity during pregnancy, and had earlier gestational age at birth. They were more likely to have ≥2 children, to use anemia medication during pregnancy, to report stillbirth/miscarriage/abortion of the last pregnancy, to have hypertensive disorder of pregnancy and gestational diabetes, and to deliver a baby with birthweight <2,500 g. They had higher household income, were less likely to have a job during pregnancy, have a spouse during pregnancy, and have a spontaneous pregnancy.

**Table 1.** Mean and prevalence of characteristics according to interpregnancy interval in the 55,203 pregnancies

	Interpregnancy interval (months)													*P* for trend

	<6	6–11	12–17	18–23	24–29	30–35	36–41	42–47	48–53	54–59	60–89	90–119	≥120	
Participants, *n*	1,587	5,349	9,388	8,526	6,721	4,903	3,901	2,967	2,257	1,807	4,724	1,755	1,318	
Age at delivery, year	28.5	29.7	30.7	31.4	32.1	32.5	32.9	33.1	33.6	33.8	34.3	35.3	37.0	<0.001
Age at first birth, year	25.2	26.7	27.4	27.7	27.9	27.8	27.7	27.3	27.3	26.9	26.1	24.6	23.1	<0.001
BMI before pregnancy, kg/m^2^	22.2	21.4	21.1	21.1	21.2	21.3	21.5	21.5	21.4	21.6	21.7	22.0	22.1	<0.001
College or higher education, %	9.7	17.2	21.7	23.2	22.7	23.7	21.3	19.2	17.7	18.5	15.1	9.4	5.1	<0.001
Smoking during pregnancy, %	11.9	6.2	3.9	3.5	3.8	4.0	4.0	4.9	5.2	5.3	7.3	10.0	15.8	<0.001
Passive smoking during pregnancy: ≥1 time/week, %	49.2	39.7	33.4	33.2	34.0	33.6	35.7	36.8	37.7	37.9	42.0	45.4	51.7	<0.001
Drinking during pregnancy, %	4.4	4.1	4.0	3.7	3.6	3.7	3.6	3.6	3.5	3.8	3.9	4.2	5.2	0.173
Physical activity during pregnancy, METs^*^min/day	307	283	272	249	245	228	225	214	209	215	210	237	270	<0.001
Household income: <4 million yen/year, %	57.9	50.4	42.8	38.8	36.6	35.7	36.0	36.4	34.9	37.9	38.0	41.2	46.1	<0.001
Having job during pregnancy, %	63.9	58.9	56.7	52.7	49.4	49.0	47.4	46.5	43.8	43.3	44.1	44.0	36.7	<0.001
Having spouse during pregnancy, %	99.0	99.1	99.4	99.4	99.1	99.0	98.8	98.6	98.8	97.4	96.6	94.1	89.0	<0.001
Number of children: ≥2 children, %	37.4	32.6	29.7	30.8	31.4	33.1	34.0	37.6	37.9	41.2	44.8	45.4	46.5	<0.001
Stress during pregnancy: K6 ≥13 points, %	4.8	3.6	2.5	2.8	2.6	2.8	2.4	2.8	3.3	3.9	3.5	4.2	5.1	<0.001
Dietary intake of iron during pregnancy, mg/day	6.7	6.8	6.9	6.8	7.0	6.9	6.9	6.8	6.8	6.8	6.8	6.8	6.8	0.500
Use of anemia medication during pregnancy, %	14.7	12.3	11.4	12.9	13.8	14.4	14.8	15.9	16.7	16.4	15.9	16.9	16.6	<0.001
Adequate intake of folate and/or folic acid, %	23.2	26.7	28.0	28.9	29.3	29.8	30.5	29.4	27.8	28.2	27.0	28.7	28.5	0.397
Spontaneous pregnancy, %	99.4	98.7	97.6	96.6	96.2	95.1	95.4	95.3	93.8	94.2	94.6	95.3	95.4	<0.001
Pregnancy outcome of last pregnancy: stillbirth, miscarriage or abortion, %	4.0	4.9	6.4	10.9	14.1	18.1	19.5	22.2	21.6	26.9	27.4	32.9	38.2	<0.001
Previous history of preterm birth, %	5.1	5.0	5.0	5.3	5.1	6.2	5.5	6.4	6.3	7.0	7.5	8.2	6.8	<0.001
Hypertensive disorder of pregnancy, %	1.6	1.7	1.6	1.6	1.9	2.1	2.4	2.2	2.2	3.3	3.5	4.2	5.8	<0.001
Gestational diabetes, %	2.1	2.2	2.1	2.5	2.4	2.7	3.2	2.7	3.5	3.2	3.8	4.3	4.8	<0.001
Birthweight: <2,500 g %	8.5	6.7	5.9	6.9	7.2	7.7	7.5	7.4	7.4	7.7	8.3	9.1	11.2	<0.001
Gestational age at birth, weeks	38.6	38.7	38.7	38.7	38.7	38.7	38.7	38.6	38.7	38.6	38.6	38.5	38.4	<0.001
Early preterm birth (22 to <34 weeks), %	1.8	0.8	0.6	0.8	0.8	0.9	0.8	1.1	1.0	0.8	1.1	1.8	2.2	<0.001
Late preterm birth (34 to <37 weeks), %	5.4	3.6	3.6	3.7	3.2	3.5	3.4	3.7	3.3	4.3	4.2	5.1	6.6	<0.001

K6, Kessler Psychological Distress Scale; METs, metabolic equivalents.

Table 2 shows the maternal age-adjusted and multivariable ORs and 95% CIs for preterm birth. Among 55,203 pregnancies, 521 early preterm births (22 to <34 weeks of gestation) and 2,094 late preterm births (34 to <37 weeks of gestation) were identified from medical record transcripts. Compared with women who had 18–23-month IPIs, both <6-month and ≥120-month IPIs were associated with increased risk of preterm birth. The multivariable ORs were 1.63 (95% CI, 1.30–2.04) for <6-month IPI and 1.41 (95% CI, 1.11–1.79) for ≥120-month IPI.

**Table 2.** Odds ratios and 95% confidence intervals for preterm birth according to the interpregnancy interval in the 55,203 pregnancies

	Interpregnancy interval, months												

	<6	6–11	12–17	18–23	24–29	30–35	36–41	42–47	48–53	54–59	60–89	90–119	≥120
**Total population**													
Number at risk	1,587	5,349	9,388	8,526	6,721	4,903	3,901	2,967	2,257	1,807	4,724	1,755	1,318
Number of cases	113	238	401	390	269	219	163	143	96	92	253	122	116
Maternal age-adjusted OR (95% CI)	1.71 (1.38–2.13)	1.01 (0.86–1.19)	0.95 (0.82–1.09)	1.00	0.86 (0.73–1.00)	0.95 (0.80–1.13)	0.88 (0.73–1.06)	1.02 (0.83–1.24)	0.88 (0.70–1.11)	1.06 (0.84–1.34)	1.11 (0.94–1.30)	1.43 (1.15–1.77)	1.77 (1.42–2.21)
Multivariable OR (95% CI)^a^	1.63 (1.30–2.04)	0.98 (0.83–1.17)	0.95 (0.82–1.10)	1.00	0.85 (0.72–1.00)	0.91 (0.77–1.09)	0.84 (0.69–1.02)	0.97 (0.79–1.18)	0.83 (0.66–1.05)	0.93 (0.73–1.19)	0.95 (0.80–1.13)	1.14 (0.91–1.43)	1.41 (1.11–1.79)

CI, confidence interval; OR, odds ratio.

^a^Adjusted for area, maternal age at delivery, body mass index before pregnancy, education levels, smoking during pregnancy, passive smoking during pregnancy, drinking during pregnancy, physical activity during pregnancy, household income during pregnancy, occupation during pregnancy, marital status during pregnancy, number of children, stress during pregnancy, dietary intake of iron during pregnancy, use of anemia medication during pregnancy, intake of dietary folate and folic acid supplementation, the latest pregnancy outcome, spontaneous pregnancy, previous history of preterm birth, hypertensive disorder of pregnancy, and gestational diabetes.

Table 3 displays the maternal age-adjusted and multivariable ORs and 95% CIs for preterm birth stratified by dietary intake of folate and supplementation with folic acid during pregnancy. The *P* for interaction between IPI and folate/folic acid intake during pregnancy on the risk of preterm birth was 0.38. Higher risks for preterm birth related to <6-month and ≥120-month IPIs compared with 18–23-month IPI were found among women with an inadequate intake of dietary folate and folic acid supplementation, while there was no association among women with an adequate intake of dietary folate and/or folic acid supplementation during pregnancy. The multivariable ORs were 1.76 (95% CI, 1.35–2.29) for <6-month IPI and 1.65 (95% CI, 1.24–2.19) for ≥120 month-IPI among women with an inadequate intake of dietary folate and folic acid supplementation during pregnancy, and 1.26 (95% CI, 0.76–2.10) for <6-month IPI and 0.88 (95% CI, 0.52–1.47) for ≥120-month IPI among women with an adequate intake of dietary folate and/or folic acid supplementation during pregnancy.

**Table 3.** Odds ratios and 95% confidence intervals of preterm birth according to the interpregnancy interval stratified by folate and/or folic acid intake in 54,685 pregnancies

	Interpregnancy interval, months												

	<6	6–11	12–17	18–23	24–29	30–35	36–41	42–47	48–53	54–59	60–89	90–119	≥120
Number at risk	1,565	5,297	9,302	8,455	6,662	4,861	3,867	2,940	2,239	1,787	4,679	1,729	1,302
**Inadequate intake of dietary folate and folic acid supplementation during pregnancy**													
Number at risk	1,202	3,881	6,699	6,008	4,707	3,414	2,687	2,077	1,617	1,283	3,418	1,232	931
Number of cases	86	163	267	260	170	151	114	101	66	59	172	76	89
Prevalence of cases, %	7.2	4.2	4.0	4.3	3.6	4.4	4.2	4.9	4.1	4.6	5.0	6.2	9.6
Maternal age-adjusted OR (95% CI)	1.85 (1.43–2.38)	1.02 (0.83–1.24)	0.94 (0.79–1.12)	1.00	0.81 (0.67–0.99)	0.99 (0.81–1.22)	0.94 (0.75–1.18)	1.08 (0.85–1.37)	0.89 (0.67–1.17)	1.00 (0.75–1.33)	1.08 (0.89–1.32)	1.31 (1.00–1.71)	2.00 (1.54–2.59)
Multivariable OR (95% CI)^a^	1.76 (1.35–2.29)	0.99 (0.81–1.22)	0.93 (0.78–1.11)	1.00	0.81 (0.66–0.99)	0.95 (0.77–1.17)	0.90 (0.71–1.14)	1.03 (0.81–1.32)	0.83 (0.63–1.11)	0.86 (0.63–1.16)	0.95 (0.77–1.17)	1.08 (0.81–1.43)	1.65 (1.24–2.19)
**Adequate intake of dietary folate and/or folic acid supplementation during pregnancy**													
Number at risk	363	1,416	2,603	2,447	1,955	1,447	1,180	863	622	504	1,261	497	371
Number of cases	20	66	117	115	88	61	40	37	29	32	76	34	21
Prevalence of cases, %	5.5	4.7	4.5	4.7	4.5	4.2	3.4	4.3	4.7	6.3	6.0	6.8	5.7
Maternal age-adjusted OR (95% CI)	1.23 (0.76–2.01)	1.02 (0.74–1.39)	0.96 (0.74–1.26)	1.00	0.95 (0.71–1.26)	0.88 (0.64–1.21)	0.69 (0.48–1.00)	0.88 (0.60–1.29)	0.96 (0.63–1.46)	1.32 (0.88–1.99)	1.24 (0.92–1.68)	1.39 (0.93–2.08)	1.12 (0.69–1.82)
Multivariable OR (95% CI)^a^	1.26 (0.76–2.10)	0.97 (0.70–1.33)	1.00 (0.77–1.31)	1.00	0.95 (0.71–1.27)	0.85 (0.62–1.18)	0.67 (0.46–0.98)	0.82 (0.56–1.22)	0.94 (0.61–1.45)	1.25 (0.82–1.90)	1.04 (0.75–1.42)	1.12 (0.73–1.71)	0.88 (0.52–1.47)

CI, confidence interval; OR, odds ratio.

^a^Adjusted for area, maternal age at delivery, body mass index before pregnancy, education levels, smoking during pregnancy, passive smoking during pregnancy, drinking during pregnancy, physical activity during pregnancy, household income during pregnancy, occupation during pregnancy, marital status during pregnancy, number of children, stress during pregnancy, dietary intake of iron during pregnancy, use of anemia medication during pregnancy, the latest pregnancy outcome, spontaneous pregnancy, previous history of preterm birth, hypertensive disorder of pregnancy, and gestational diabetes.

## DISCUSSION

In a large birth cohort study, we found that both <6-month and ≥120-month IPIs were associated with increased risk of preterm birth. For <6-month and ≥120-month IPIs, the risks of preterm birth were 1.4- to 1.6-fold higher than those with 18–23-month IPI. The interaction between IPI and folate/folic acid intake on the risk of preterm birth was not statistically significant; however, higher risks of preterm birth were confined to women with inadequate intake of dietary folate or those with folic acid supplementation during pregnancy.

Our findings are consistent with those of previous studies.^4^^,^^5^ A United States study using vital statistics data among approximately 170,000 singleton livebirths found that, compared with 18–23-month IPI, both 0–5-month and ≥120-month IPIs were associated with 1.4- to 1.5-fold increased risk of preterm birth (livebirths at <37 weeks of gestation).^4^ Another United States study using birth certificate data of approximately 430,000 singleton livebirths reported that both 0–5-month and ≥120-month IPIs were associated with higher risk of preterm birth (live births at <37 weeks of gestation), regardless of race; multivariable-adjusted ORs compared with 18–23-month IPIs according to race were 1.3 (95% CI, 1.2–1.4) for white women with 0–5-month IPI, 1.4 (95% CI, 1.3–1.5) for white women with ≥120-month IPI, 1.2 (95% CI, 1.1–1.3) for black women with 0–5-month IPI, and 1.3 (95% CI, 1.2–1.4) for black women with ≥120-month IPI.^5^ Regarding the Asian population, a Taiwanese retrospective cohort study reported that <12-month IPI led to a 1.3-fold increased risk of preterm birth (live birth at 20–36 weeks of gestation) compared with ≥12-month IPI^24^; however, a detailed analysis of the association between the fine categories of IPI and risk of preterm birth was not conducted.

The observed higher risk of preterm birth associated with short IPI may be explained by the maternal nutritional depletion hypothesis, in which lactation and insufficient time to recover from the physiological stress of the previous pregnancy adversely affect maternal nutritional status.^25^^,^^26^ Maternal folate concentrations remained lower after delivery, and 20% of women had folate deficiency 6 months after delivery.^11^ Pregnant women with short IPI have an increased risk of folate deficiency—a risk factor for preterm birth.^27^^–^^29^

Nevertheless, it is unclear why a long IPI increased the risk of preterm birth. Zhu et al offered two hypotheses about the mechanisms for this.^4^ The first is the “physiological regression hypothesis,” by which pregnancy causes anatomical, physiological, and biochemical adaptations in the reproductive system that gradually return to baseline conditions after delivery. When women are not pregnant or have not delivered a baby for a long time, the characteristics of reproductive system may become similar to those of women who become pregnant for the first time. The second hypothesis is that a long IPI may be caused by infertility-related factors, thereby increasing the risk of preterm birth.^30^

We found a higher risk of preterm birth related to <6-month and ≥120-month IPIs among women with inadequate intake of dietary folate and folic acid supplementation during pregnancy. Folate is a cofactor for single-carbon transfers in the metabolism of nucleotides and amino acids; folate deficiency caused higher homocysteine levels,^31^^–^^34^ which is a risk factor for atherosclerosis^35^ and thrombosis.^36^^,^^37^ For pregnant women, lower folate levels might affect placentation during pregnancy. A previous study reported that pregnant rats without folate supplementation tended to have lower plasma folate levels, higher plasma homocysteine levels, and decreased placental DNA methylation and placental weight.^38^ For pregnant women, lower blood folate levels (≤9.2 nmol/L) during pregnancy (median: 13.2 weeks of gestation) were associated with lower placental weights and higher risks of preterm birth (<37 weeks of gestation).^27^

The strengths of our study include its prospective design and the large sample size in a national birth cohort. We examined associations between fine categories of IPI and risk of preterm birth after extensive adjustment for potentially confounding factors.

Our study has a few limitations that need mentioning. First, we could not consider pregnancy following miscarriage to calculate IPI. A recent meta-analysis of ten studies indicated that a <6-month IPI following miscarriage was not associated with a higher risk of preterm birth.^39^ Second, information regarding supplementation with folic acid during pregnancy was collected from self-reported questionnaires. Nevertheless, a previous study reported that Pearson’s correlation coefficient for comparing questionnaire data with interview responses was 0.76 for supplementation with folic acid.^40^ Third, due to the lack of information on presence or absence of labor, which is necessary to define spontaneous preterm birth,^41^ we could not discriminate preterm birth as spontaneous or indicated preterm birth. However, a previous study showed that <6-month IPI was associated with an increased risk of both spontaneous and indicated preterm birth (<37 weeks of gestation) than was 18-month IPI.^42^

In conclusion, both <6-month and ≥120-month IPIs were associated with increased risk of preterm birth in pregnant Japanese women. The interaction between IPI and folate/folic acid intake on risk of preterm birth was not statistically significant; however, higher risks of preterm birth related to <6-month and ≥120-month IPIs were found in women with inadequate intake of dietary folate and folic acid supplementation during pregnancy, suggesting that adequate intake of dietary folate or folic acid supplementation during pregnancy may contribute to decreased risk of preterm birth related to IPI.
